# Initiatives and partnerships in an Australian metropolitan obesity prevention system: a social network analysis

**DOI:** 10.1186/s12889-021-11599-7

**Published:** 2021-08-12

**Authors:** Krysten Blackford, Justine E. Leavy, Abbie-Clare Vidler, Dan Chamberlain, Christina Pollard, Therese Riley, Megan Milligan, Jonine Jancey

**Affiliations:** 1 School of Population Health, Curtin University, Perth, Australia; 2 Collaboration for Evidence, Research and Impact in Public Health, Curtin University, Perth, Australia; 3 The Australian Prevention Partnership Centre, Sax Institute, Haymarket, Australia; 4 Centre for Social Impact, UNSW, Sydney, Australia; 5 East Metropolitan Health Service, Perth, Australia

**Keywords:** Social network analysis, Obesity prevention, Health promotion, Systems thinking, Partnerships, Non-communicable disease

## Abstract

**Background:**

Limited resources make prevention of complex population-level issues such as obesity increasingly challenging. Collaboration and partnerships between organisations operating in the same system can assist, however, there is a paucity of research into how relationships function at a local level. The aim of this study was to audit initiatives, explore networks, and identify potential opportunities for improving the obesity prevention system in a Health Service area of Western Australia (WA).

**Methods:**

A mixed-methods study was undertaken in a metropolitan Health Service in Perth, WA in 2019–20. Structured face-to-face interviews (*n* = 51) were conducted with organisations engaged in obesity prevention, to identify prevention initiatives and their characteristics using a Systems Inventory tool. The Research Team identified the 30 most active organisations during the Systems Inventory, and an online Organisational Network Survey was administered to explore: relationships across six domains; partnership duration; frequency of interaction with other organisations; barriers to implementation; and key contributions to obesity prevention. Descriptive statistics were used to summarise barriers, contributions and Systems Inventory data. Organisational Network Survey data were analysed using social network analysis through UCINET 6 for Windows and Netdraw software. Whole network and cohesion scores were calculated: average degree; density; diameter; and degree centralization. Core-periphery analysis was conducted to identify densely connected core and sparsely connected periphery organisations.

**Results:**

The Systems Inventory identified 189 unique prevention initiatives, mostly focusing on individual-level behaviour change. Fifty four percent (*n* = 15) of the Organisational Network Survey respondent organisations and most core organisations (67%, *n* = 8) were government. The information and knowledge sharing network had a density of 45% indicating a high level of information and knowledge exchange between organisations. The lowest densities were found within the receiving (3.3%), providing (5.5%) and sharing (5.6%) funding networks, suggesting that these formal relationships were the least established.

**Conclusion:**

Applying a systems thinking lens to local obesity prevention revealed that initiatives conducted focused on individual-level behaviour change and that collaboration and communication between organisations focused on information sharing. Capturing the extent and nature of initiatives and the way partnerships operate to improve obesity prevention can help to identify opportunities to strengthen the networks.

**Supplementary Information:**

The online version contains supplementary material available at 10.1186/s12889-021-11599-7.

## Background

Each year 36 million people die from preventable non-communicable disease (NCD), accounting for 63% of global deaths [1]. Obesity is an important risk factor for NCDs with its prevalence influenced by a complex mix of biological, behavioural and environmental factors [2]. More than 1.9 billion people worldwide were classified as overweight and/or obese [3] and high body mass index (BMI) attributed to four million deaths globally in 2016 [2, 4]. In Australia, two-thirds of adults were classified as overweight or obese in 2018 [5]; with physical inactivity, poor diet, and overweight and obesity independently contributing to the disease burden (2.5, 7.3, and 8.4% respectively) [6]. It is estimated that the direct and indirect costs associated with obesity will increase to $87.7 billion by 2025 if no further preventive action be taken in Australia [7].

Evidence suggests that a collaborative, coordinated, multi-strategy effort is needed to address the complex determinants of obesity. Yet there is a tendency for prevention organisations to work in silos, which limits interaction between groups or sectors and creates competition for limited funding and resources [8–12]. Insufficient funding may also lead to competitive program design, with organisations at times seeking to implement isolated prevention strategies to justify their influence on health and ongoing funding [13]. Stand-alone programs have the potential to use limited resources inefficiently and oversaturate the prevention space with duplicative initiatives [13, 14]. Accordingly, with increasingly limited resources and a perceived duplication of initiatives, it is timely to investigate more synergistic approaches to address obesity.

Systems thinking has emerged within public health as an approach to explore complex problems and the interconnected factors that contribute to the prevention efforts within a whole system [15–17]. The adoption of systems thinking in public health is in recognition of its ability to inform investments and actions that address complex problems in specific domains, including obesity prevention [17]. This approach can determine the dynamic interrelationships between system components, facilitating a better understanding of the shifting influences that exist and affect a system’s operation [18, 19].

The systems approach provides tools to systematically synthesise existing data, expose gaps, inform priority setting, and identify leverage points for improving the operation of the system [20–22]. Suggested methods for this type of approach include: investigating system attributes; identifying relationships between attributes or actors; and pinpointing the key interactions in the system that facilitate or hinder activities [17]. Social network analysis (SNA) is a tool commonly used to develop an understanding of the opportunities and barriers faced, and roles organisations play within a prevention system [15, 23, 24]. The focus of SNA is on relationships within a network rather than characteristics of individuals or organisations that comprise the network [25]. This enables investigation of the types of relationships that exist (e.g., informal versus formal) and provides an understanding of network operations and the roles of key actors or organisations [26].

Understanding initiatives and collaborations between organisations is particularly important in obesity prevention, considering the role of partnerships for addressing such multifaceted issues [1, 27, 28]. The prevention system in Australia is faced with funding barriers and increasing demand for quality and efficiency, which makes prevention of population-level issues such as obesity increasingly challenging [29]. Dispersed efforts can be addressed through collaboration and partnerships between organisations operating in the same space [29]; however, there is a paucity of research into how these relationships function within the obesity prevention system. By understanding prevention networks and the ways in which partnerships operate, current efforts to improve health can be better understood and potentially strengthened [30].

A systems approach was undertaken to understand NCD prevention initiatives in a small number of communities in Australia during the Prevention Tracker project [29]. These studies explored how each community worked to prevent NCDs by identifying prevention initiatives and collaborations by undertaking SNA and community consultations. Findings indicate that hidden roles may create and exacerbate barriers to cross-sector coordination and suggest that collaborations and communications are necessary to strengthen NCD prevention systems [31]. The present study adapted the methods used during this previous work to gain a better understanding of the obesity prevention system within a newly formed Health Service, which was established in 2016 with the aim of maintaining and improving the health and wellbeing of the 725,000 residents in its catchment area. The Health Service is a government organisation overseeing a network of health facilities and services across 13 local government areas (LGAs), and sought to understand the organisations and networks that operate within the Health Service area for obesity prevention.

The aim of this study was to identify the prevention initiatives and explore the organisational networks across the Health Service area to identify potential opportunities for health promotion investments to improve obesity prevention efforts. Obesity prevention initiatives of interest included health promotion or public health interventions addressing physical inactivity and/or poor diet at a community or population level. The specific research objectives were to: 1) audit the physical activity, nutrition and obesity prevention initiatives taking place in the area; 2) identify the most influential stakeholders and collaborations; and 3) identify opportunities to make system improvements.

## Methods

This mixed methods study design was adapted from previous studies undertaken in Australia [29, 32]. The study was conducted in two phases: 1) a Systems Inventory to identify physical activity, nutrition and obesity prevention initiatives in the area; and 2) an Organisational Network Survey of a purposeful sample of key organisations to explore networks and collaborations. The full methods are described in detail in the study protocol paper [33]. Ethical approval was obtained from the Curtin University Human Research Ethics Committee (approval number HRE2017–0862).

### Systems inventory

#### Participants and sampling

The Advisory Group, comprising researchers (*n* = 4), Health Service health promotion staff (*n* = 3), and experts in physical activity, nutrition and obesity prevention (*n* = 2), used Nominal Group Technique [34] to generate a list of organisations likely to undertake relevant prevention activities in the study area. Organisations were included if they were responsible for delivering obesity prevention initiatives in the study area that were population- or community-based and ongoing in nature. Organisations were excluded if they were clinical and/or responsible for delivering obesity initiatives that focused on treatment at an individual level rather than prevention. This process provided consensus on the key organisations and contacts to be included in the sample. Potential organisations were invited to participate in a face-to-face interview to outline obesity prevention initiatives in their area. Organisations nominated staff to be interviewed based on their knowledge of, and experience with the organisation’s obesity prevention initiatives, and study participants received a study information sheet and provided informed consent prior to commencing the interview. To ensure that all relevant organisations were included in the inventory, a snowball sampling technique was used whereby interviewed participants nominated other relevant organisations in the study area [35].

#### Data collection

The Systems Inventory instrument [36] was based on a previously trialed inventory [37–39] used to collect data on local prevention programs and activities within specific communities for the Prevention Tracker project [29]. The instrument was adapted for the current study, reviewed by the Research Team and Health Promotion Officers to confirm face and content validity [40]. The final instrument captured information about each organisation and each initiative delivered, including the objectives and strategies; types and durations of each initiative; collaborating partners; target population; settings and locations; evaluation; and funding details (refer to Additional File 1). A custom-built Microsoft Access database was used to record the Systems Inventory prevention initiatives using a structured questionnaire, which included a series of dichotomous (yes/no), multiple choice and frequency questions about current prevention initiatives undertaken by each organisation.

Initiatives were included if they were: a) a current policy, regulation or program; b) conducted in the study area; c) composed of more than one session (to indicate an ongoing nature); d) population- or community-based; and e) aimed at physical activity, nutrition or obesity prevention. Initiatives were excluded if they were: a) delivered by alternative therapists based on Australian Health Practitioner Regulation Agency [41] guidelines; b) pharmacological interventions (including special purpose dietary supplements); c) one-on-one interventions (e.g. practitioner delivered individual focused programs; and d) not comprising a core component (at least 75%) focusing on physical activity, nutrition or obesity prevention. Each participant was asked if they would be willing to participate in a follow-up survey.

Systems inventory prevention initiatives were collected during face-to-face structured interviews. Pilot interviews tested the recording procedures with health professionals (*n* = 10) not involved in the study and six health promotion officers from the Health Service, and modifications were made to wording and content to ensure usability, suitability and comprehension. Eight trained Health Promotion Officers from the Health Service paired up to conduct the face-to-face interviews, which took one to two hours to complete.

### Organisational network survey

#### Participants and sampling

Guided by previous studies [29, 42], the Research Team identified the 30 organisations from the Systems Inventory data deemed to be the most active in obesity prevention in the study area based on the number and types of initiatives delivered. Members of the sample were contacted via telephone to invite participation, and participants were sent a study information sheet and Qualtrics survey via email. Participants provided informed consent via a check box prior to commencing the survey. If there was no response after one week, researchers called back and offered assistance to complete the survey. For data to be considered reliable a response rate of 75% was required [43].

#### Data collection

A roster of the 30 organisations was generated, and data were collected via an online survey adapted from previous studies [29, 42] and inputted into Qualtrics [44] (refer to Additional File 2). Participants were asked to answer a series of network questions for each of the identified organisations (*n* = 30), which asked about the: relationship with each organisation across several domains (share information or knowledge; share resources; engage in joint planning or run joint programs; receive funding; provide funding; share funding or apply for joint funding) using a scale (high, medium, low, or none). Additional survey questions included the following: barriers to implementing obesity prevention in the study area from a list provided (*n* = 21); partnership duration (< 6 months, 6 months to 2 years, > 2 years) with each organisation; the most important contribution their organisation makes to obesity prevention; and demographic and organisational characteristics.

The survey was reviewed by the Advisory Group to establish face and content validity [40]. A group interview was conducted with health promotion staff (*n* = 6) from an organisation who had no involvement in the research, to check for comprehension. The final instrument was trialled with Health Promotion Officers (*n* = 10) working in the Health Service.

### Data analysis

To address study objective 1 (audit the physical activity, nutrition and obesity prevention initiatives taking place in the area), descriptive statistics were used to summarise the Systems Inventory data. To address study objective 2 (identify the most influential stakeholders and collaborations) the barriers and contributions identified by each organisation during the Organisational Network Survey were analysed using descriptive statistics, along with analysis of the Organisational Network Survey data using SNA. Descriptive statistics were analysed using SPSS Statistics Package 25 [45], and SNA data were mapped and analysed using UCINET 6 for Windows [46] and Netdraw software [47]. Each of the relationship domains (share information or knowledge; share resources; engage in joint planning or run joint programs; receive funding; provide funding; share funding or apply for joint funding) were treated as individual networks. The individual networks were also combined/flattened [48] to create a composite network. Networks were mapped visually and the analysis examined the role of organisations in the network relative to others by using whole network and cohesion scores. Structural properties that were investigated included: *average degree* (the number of ties an organisation has); *density* (the proportion of possible ties between organisations); *diameter* (the shortest path between the two organisations furthest from each other); and *degree centralization* (tendency of the network to focus connections on a single organisation) [23, 26, 49–51]. Organisation characteristics were used to determine the basis of clustering to enable the identification of potential organisational collaborations and gaps in the service delivery system across the study area.

Core-periphery analysis was conducted using a composite of the network across the six relationship domains to identify densely connected core-nodes and sparsely connected periphery-nodes [29, 52]. Core organisations were characterised by dense connections with other members of the core, and peripheral organisations were characterised by relatively less dense connections with other members in this group [29]. Non-respondents were excluded from the core-periphery analysis. Nodes were de-identified for presentation.

## Results

### System inventory

The Systems Inventory results address study objective 1: audit the physical activity, nutrition and obesity prevention initiatives taking place in the area. Fifty-one staff from government and non-government organisations participated in the Systems Inventory interviews. A total of 189 nutrition, physical activity and obesity prevention initiatives were identified. A summary of the objectives, strategies, target groups, setting and funding sources of the initiatives is presented in Additional File 3.

The majority of initiatives were physical activity focused (50%, *n* = 95), 35% were nutrition focused (*n* = 66) and 15% obesity prevention focused (*n* = 28). Overall, the most common objectives were: influence attitudes (87%, *n* = 164); behaviour change (86%, *n* = 162); increase knowledge (85%, *n* = 160); build skills (82%, *n* = 156); and raise awareness (80%, *n* = 152). The least common objectives were: influence changes to the built environment (32%, *n* = 60); advocate for change (32%, *n* = 17); and to develop regulation (9%, *n* = 18).

The most common strategies to implement initiatives were communication or education based: online communications (79%, *n* = 150); printed resources (76%, *n* = 144); social media (70%, *n* = 132); group education sessions (67%, *n* = 127); and unpaid media (61%, *n* = 116). Partnership development was a strategy in over 70% of initiatives. Implementing policy and guidelines (24%, *n* = 45); paid media (20%, *n* = 38); and school curriculum (10%, *n* = 18) were the least common strategies.

The primary target groups were varied, from everyone (18%, *n* = 34), adults aged 50 years or more (10%, *n* = 19), to employees (9%, *n* = 17). Community centres (39%, *n* = 73), leisure centres (30%, *n* = 57) and workplaces were the main settings initiatives occurred in. Early childhood settings were the least commonly used, (e.g. play groups (4%, *n* = 7) and day care centres (3%, *n* = 5)). Thirty initiatives (16%, *n* = 30) operated state-wide.

Government was the most commonly reported source of funding, and included state (14%, *n* = 26), local (12%, *n* = 23), or federal government (2%, *n* = 4). State government funded more obesity prevention initiatives (21%, *n* = 6) compared with nutrition or physical activity.

### Organisational network survey

The Organisational Network Survey results address study objective 2: identify the most influential stakeholders and collaborations.

#### Organisation characteristics

Twenty eight of the 30 organisations invited to participate completed the Organisational Network Survey (participation rate = 93%). The two organisations that did not respond were coded as non-respondents and included in the SNA. All organisations were involved in implementing obesity prevention, physical activity or nutrition initiatives in the study area. Just over half (54%, *n* = 15) of respondents were government organisations (including local and state government organisations) and the remainder (46%, *n* = 13) were non-government (including philanthropic and not-for-profit health services). The mean number of full time equivalent staff per organisation was 35 (range 1–400). The mean operation length was 60 years (range 3–150 years). On average, each organisation was aware of 15 of the 29 other organisations listed in the organisation list. Long-term partnerships of two or more years were the most frequently reported (*n* = 222), followed by no relationship (*n* = 125), medium-term partnership (6 months to 2 years) (*n* = 76) and short-term partnership (< 6-months) (*n* = 18).

#### Barriers and contributions

The six most common barriers to implementing initiatives identified were: limited funding (79%, n = 22); limited staffing (79%, n = 22); no formal health policy (32%, *n* = 9); a lack of political feasibility or amenability to prevention and health promotion (25%, *n* = 7); insufficient collaborations and partnerships (21%, *n* = 6); and limited in-kind resources (21%, *n* = 6). The five highest ranked contributors to obesity prevention were having paid staff; funding; program support and coordination; program development and evaluation; and community connections. The five lowest ranking contributions were: developing health policy; expertise other than health; advocacy; volunteer staff; and information technology or web resources.

#### Networks

**Fig. 1:**
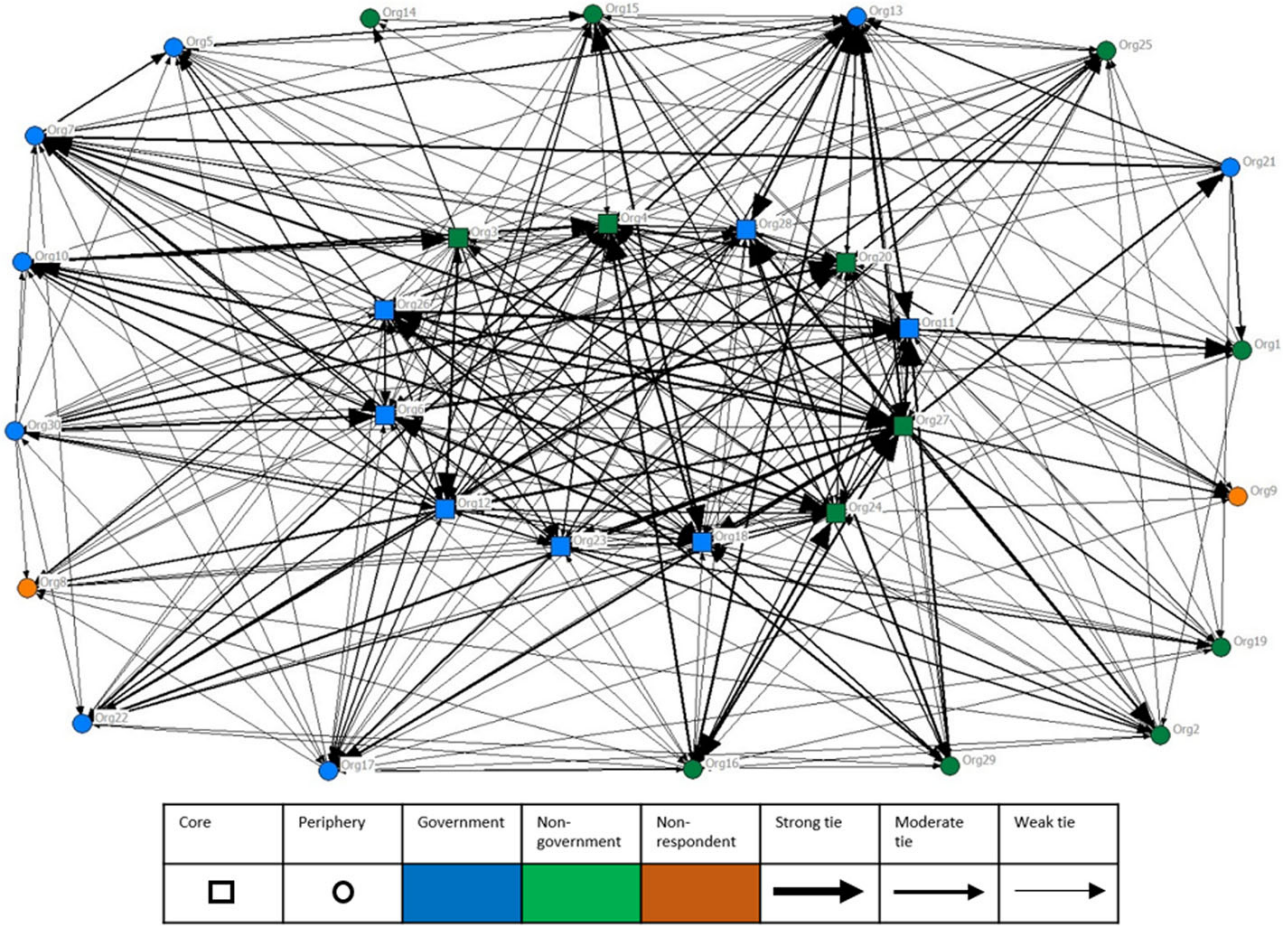
Information and knowledge sharing between organisations in the Health Service area. This figure shows the relationships between core (square) organisations at the centre of the figures, and the periphery (circle) organisations, as well as government (blue) and non-government organisations (green). Strength of relationships is shown by the lines, with darker lines representing stronger relationships between organisations. The non-respondents are shown in orange

**Fig. 2:**
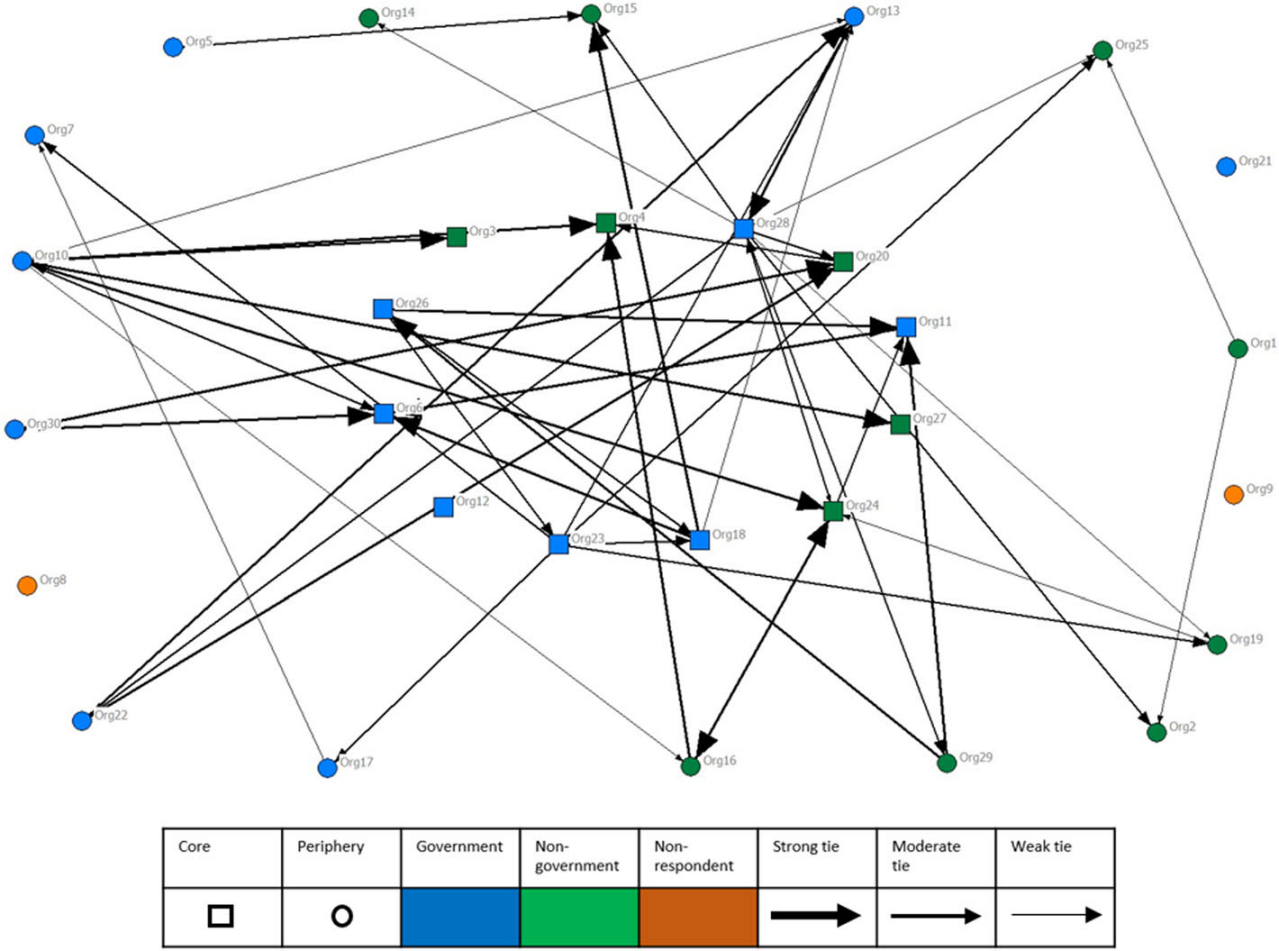
Shared funding and applications for joint funding between organisations in the Health Service area. This figure shows the relationships between core (square) organisations at the centre of the figures, and the periphery (circle) organisations, as well as government (blue) and non-government organisations (green). Strength of relationships is shown by the lines, with darker lines representing stronger relationships between organisations. The non-respondents are shown in orange

The core-periphery analysis revealed 43% of organisations (*n* = 12) were in the network core, including eight government organisations. Figure 1 depicts the information and knowledge sharing between organisations, and Fig. 2 depicts the shared funding and applications for joint funding between organisations. These figures show the relationships between core (square) organisations at the centre of the figures, and the periphery (circle) organisations, as well as government (blue) and non-government organisations (green). Strength of relationships (nominated as ‘high’ in the Organisational Network Survey network questions) is shown by the lines, with darker lines representing stronger relationships between organisations. Figure 1 (information and knowledge sharing) represents a densely connected network, and Fig. 2 (shared funding and applications for joint funding) represents a sparsely connected network. The non-respondents are shown in orange.

Table 1 provides a summary of the cohesion metrics across all relationships. The information and knowledge sharing network had a density of 45% indicating a high level of information and knowledge exchange between organisations. The average degree for this network was 13.13 which represents the number of ties an organisation has; and the degree centralization was moderate at 0.59, indicating there is a tendency for the network to focus connections on a single organisation. Overall it can be suggested that information travels quickly between organisations in this network.

**Table 1:** Cohesion metrics across all relationships

	Composite network	Sharing information or knowledge	Sharing resources	Joint planning, or running joint programs	Receiving funding	Providing funding	Sharing funding
Average Degree	13.23	13.13	9.10	9.10	0.97	1.60	1.63
Density	0.46	0.45	0.31	0.31	0.03	0.06	0.06
Diameter	3	3	4	4	2	6	6
Degree Centralization	0.58	0.59	0.59	0.59	0.23	0.35	0.31

The networks for sharing resources, engaging in joint planning or running joint programs, and receiving, providing and sharing funding were progressively less dense and less connected than the information and knowledge sharing network. The lowest densities were found within the receiving funding (3.3%), providing funding (5.5%) and sharing funding (5.6%) networks, suggesting that funding relationships are the least well established in the network. These networks also had lower average degrees relative to other networks, indicating fewer relationships between organisations in terms of funding relationships.

The structure and cohesion metrics for the composite network suggest that overall there were strong connections between organisations in the study area. The density was 46% indicating that nearly half of the potential connections in the network are present. An average degree of 13.233 shows that on average, each organisation is connected to almost half of the total organisations in the network. The degree centralization was moderate at 0.583, indicating there is a tendency of the network to focus connections on a single organisation, which is a similar finding to the information and knowledge sharing network.

## Discussion

This study utilised a systems thinking framework and tools to explore the obesity prevention initiatives and networks in the 13 LGAs comprising one metropolitan WA Health Service area. The inventory identified 189 nutrition, physical activity and obesity prevention initiatives delivered across the study area. The SNA identified strengths in connection and interaction between organisations across information and resource sharing domains, and suggested that joint funding and planning were not as well established in the network. The research findings provide insights into opportunities to enhance prevention strategies, collaborations and intended outcomes across a Health Service area.

### Local physical activity, nutrition and obesity prevention activities

Most prevention initiatives in this study focused on behaviour change strategies at the individual level. The 189 initiatives were predominantly aimed at raising awareness, and changing attitudes, knowledge, behaviour and skills using media and education strategies. Few initiatives focused on influencing changes to the built environment, advocating for change, or developing policies and regulations. An individual’s ability to meet dietary and physical activity guidelines to maintain a healthy body weight is influenced by their knowledge and skills as well as the built environment (e.g., access to recreation, available food options) [30]. Research suggests that a comprehensive approach is needed to address the determinants of obesity [53, 54], and individual-level single solution behaviour programs are likely to be difficult to sustain and may have limited success in the long term [30, 55–57]. Consideration of approaches beyond individual-level behaviour change will better meet the diverse needs of a population, and create an environment supportive of positive, long-term health outcomes [28, 58, 59].

Environmental change to enhance health behaviours is challenging, especially in the complex area of obesity. Achieving long-term change requires overarching governance, shared vision, clear leadership, communication, trust, capacity and performance indicators [24, 44, 60]. Where uncoordinated action occurs, local communities may receive inefficient and ineffective initiatives that are unevenly distributed, with organisations competing for limited resources [44]. However, strategic priority setting and funding can encourage consideration of the social, environmental and behavioural determinants of health [2], with interventions encompassing a broader range of objectives and strategies to strengthen the effectiveness of prevention efforts [14]. This process requires a strategic and coordinated approach to achieve changes across a network, ideally with the support of central organisations.

### Influential stakeholders and collaborations

Organisations in the present study were well connected and interacted with one another across information and resource sharing domains; however, joint funding and joint planning were not well established across the network. This is consistent with previous research [29] that has found informal networks (e.g., information and resource sharing) are densely connected, while formal networks such as joint funding applications and joint planning have weaker connections; and suggests that some of the network connections may not be reaching their full potential. Informal networks are likely to move to more formal structures over time [61]; however, this can be challenging for self-organising networks operating in systems with limited strategic oversight and diffuse governance [12].

Although formal collaborations and partnerships are recommended as a way of sharing limited resources and reducing duplication of initiatives, barriers can exist at the funding and organisation levels [29]. The present study found that limited funding, staffing, and in-kind resources all present barriers to prevention initiatives. Collaborations, particularly formal collaborations, come with time and human resources costs, and these costs need to be offset by the benefits stemming from collaborations [12]. Some funding bodies will mandate collaborations between organisations which can lead to “partnerships on paper” in order to meet funding requirements, even though funded collaborations require a high level of commitment and do not always result in better collaborations [62]. Organisations may find it difficult to align their priorities when delivering joint initiatives [50, 63], and funding and political cycles can create short-term relationships between organisations making it difficult to maintain collaborations long term [50]. Organisations applying for the same funding may also see each other as competitors which can create tensions [29]. A range of strategies may address some of these barriers, such as: rewarding long-term investment in collaborative practice by providing funding to existing relationships [26]; local organisations building on existing infrastructure of informal networks when demonstrating capacity to meet funding requirements and deliver services; and funding bodies recognising local initiatives rather than imposing top-down models of effective partnerships [29].

The core-periphery analysis provided additional information on the study’s network structure. SNA can provide researchers with insight into whether organisations are working together, and the core-periphery analysis goes one step further by answering questions such as: “Who is in the best position to benefit, to lead or to bring the network together? Could opportunities be distributed more equitably? Who should be connecting with whom, considering their role and resources?” [60]. Universally, core organisations have been found to provide strategic direction and expertise across the network having rich connections that can be used to access resources and information compared to periphery organisations. Core organisations are active in overseeing or knowing about the initiatives of others and supporting effective functioning of the system, while peripheral organisation are focussed on delivering initiatives [60].

Core-periphery structure of a network can be further explored in the context of the complementary contributions made by government and community organisations. A previous study investigating the contributions to prevention by different types of organisations [64] found key differences between LGAs and community health organisations. The perceived contributions to prevention by LGAs in the previous study were: cross-sectoral relationships; influence on the structural, cultural, social and physical determinants of health; and a population-wide reach. LGA functions are diverse (urban planning, community wellbeing, leadership, lobbying to state government) and they can mandate structural change within community and have influence over businesses, residents and workplaces. Conversely, community health organisations and NGOs were seen to contribute to local prevention through: community development approach and philosophy; community connections; and knowledge and expertise. Practitioners becoming more aware of the key contributions made by other types of organisations in the network may be able to encourage collaborations by highlighting and building on the complementary nature of these contributions.

The terms “core” and “periphery” are not intended to imply that any type of organisation is more valuable or important than another, but to assist in understanding of the role and density of each type of organisation in a network. There are limitations when the density of relationships at the core is high, such as the risk of organisations becoming “closed-off” to new information and operating under the usual way of working [60]. Provision of additional resources and opportunities for those organisations in the periphery and increasing practitioner awareness of the benefits and limitations of the core-periphery structure in the local system have been shown to distribute resources and collaborations more evenly [60].

### Opportunities to make system improvements

This study highlights the complementary nature of the contributions made by different organisation types operating in a system. It provides an opportunity to consider these findings and identify opportunities to continue current practices or modify these practices to enhance prevention strategies and intended outcomes. Opportunities include: consideration of prevention initiatives that emphasise environmental supports [30]; encouragement of formal and informal information sharing and joint planning across the system so that limited resources are best utilised [61]; advocacy for funding bodies to reward long-term collaborative practice by providing funding to existing collaborations and recognition of local initiatives [29]; increasing practitioner awareness of the contributions made by all organisations in the network to encourage collaborations [60]; recognition that core organisations play a vital role in transmitting information, with the ability to oversee and understand what activities are occurring [60]; and more engagement with sectors outside of health to enable prevention work to occur outside of the usual bureaucratic and political silos [31].

### Strengths and limitations

The use of Health Promotion Officers from the Health Service to recruit and collect data ensured a certain level of trust with organisations in the community. Data were collected quickly without relying on significant financial resources. An additional strength is use of the Systems Inventory as a baseline measure that can be used for planning and assessing the prevention response. It should be noted that the study did not assess the quality and impact of initiatives and this is recommended for future research endeavours. Additionally, the study does not comment on changing organisational positions in the network across time [60]; however, there is currently a dearth of evidence regarding the methods and tools to capture this longitudinal type of data [17].

The study recruited via “word-of-mouth” and it is possible that some organisations and their initiatives may have been omitted. Furthermore, there may have been a lack of shared understanding of prevention and the systems thinking concepts and language between practitioners and researchers. Previous research has shown that even those in senior positions who consider themselves to be systems thinkers are often considering the health system specifically, rather than the whole system that would be considered by systems scientists [65]. In addition, the identification of the core network organisations were based on defined factors (e.g. share information or knowledge; share resources; engage in joint planning or run joint programs; receive funding; provide funding; share funding or apply for joint funding), therefore should other factors or organisations be examined it would potentially reveal a different core-periphery structure [60].

## Conclusion

This study provides a snapshot of the number and types of initiatives, their organisational and other characteristics and social networking components of an obesity prevention system in one Health Service area. The findings add to the small but growing body of literature on the use of systems thinking tools to explore how local communities work to prevent obesity and NCDs. Findings provide insights into the prevention initiatives, organisational structure and collaborations between organisations. By understanding prevention initiatives and the ways in which partnerships operate, efforts to improve obesity prevention can be discussed, better understood and potentially strengthened.

## Supplementary Information

**Additional File 1: **Systems Inventory Instrument**.** The Systems Inventory instrument captured information about each organisation and each initiative delivered in the study area, including the objectives and strategies; types and durations of each initiative; collaborating partners; target population; settings and locations; evaluation; and funding details. The data were collected using a custom-built Microsoft Access database.

**Additional File 2: **Organisational Network Survey. Organisational network data were collected via an online survey using Qualtrics. Participants were asked to answer a series of network questions for each of the identified organisations (*n* = 30), which asked about the: relationship with each organisation across several domains (share information or knowledge; share resources; engage in joint planning or run joint programs; receive funding; provide funding; share funding or apply for joint funding) using a scale (high, medium, low, or none).

**Additional File 3: **Prevention system inventory (*n* = 189): This table presents a summary of the objectives, strategies, target groups, setting and funding sources of the initiatives collected during the Systems Inventory.

## Data Availability

The datasets generated and analysed during the current study are not publicly available due to confidentiality of organisation information but de-identified data are available from the corresponding author on reasonable request.

## Abbreviations

LGA: Local government areas
NCD: Non-communicable disease
SNA: Social network analysis
WA: Western Australia
