# Supplementary material for: Initiatives and partnerships in an Australian metropolitan obesity prevention system: a social network analysis
Source: BMC Public Health. 2021 Aug 12;21:1542. doi: 10.1186/s12889-021-11599-7 (PMC8359547; doi:10.1186/s12889-021-11599-7)
Supplement: Supplementary file 1 — Additional File 1: Systems Inventory Instrument. The Systems Inventory instrument captured information about each organisation and each initiative delivered in the study area, including the objectives and strategies; types and durations of each initiative; collaborating partners; target population; settings and locations; evaluation; and funding details. The data were collected using a custom-built Microsoft Access database. [file 12889_2021_11599_MOESM1_ESM.docx]

**Additional Files**

**Additional File 1**

**Title of instrument: Systems Inventory Instrument**

**Description of instrument:** The Systems Inventory instrument captured information about each organisation and each initiative delivered in the study area, including the objectives and strategies; types and durations of each initiative; collaborating partners; target population; settings and locations; evaluation; and funding details. The data were collected using a custom-built Microsoft Access database.

|  | Question  number |  | Initial audit  Audit further information  Network analysis | Interviewer to complete post interview and comments for database |
| --- | --- | --- | --- | --- |
| Interviewer Information |  | The following information is to be complete by the person who will be conducting the interview:  **Name of the person:** | First name  Last name | Textbox field  Textbox field |
|  |  |  |  |  |
| Preamble |  | **Interviewer – please select one of the two options below:**   1. **If contact has already been established:**   Thank you for agreeing to participate in our **Prevention Tracker** audit of health promotion programs to improve nutrition, physical activity and reduce overweight and obesity.  To remind you of the reason for our meeting, we are interested in understanding what physical activity, nutrition and overweight and obesity programs are being conducted in your area, as we want to form a picture of these **Prevention Programs** in our local community. The results will help us work with you and others to better plan and develop services to meet local needs. The findings of the audit will be provided to you once they are collated.  On average the survey takes [**about 30-40 minutes]**. The answers from all people interviewed will be combined and your responses will remain confidential. Participation in the survey is voluntary. You may withdraw from the survey at any time or refuse to answer any questions if you wish **(please refer to information sheet for additional information).**   1. **If contact has NOT already been established:**   Hello. I'm [**name of interviewer**] from [Organisation Name]. I am making contact with you as we are interested in understanding what physical activity, nutrition and overweight and obesity programs are being conducted in your area, as we want to form a picture of these **Prevention Programs** in our local community. These findings will help us work with you and others to better plan and develop services to meet local needs. The findings of the audit will be provided to you once they are collated.  Therefore, we need to speak to someone who knows about the types of physical activity, nutrition and overweight/obesity programs that are being conducted in [**your area**]. Would that be you or somebody else?  On average the survey takes **[about 30-40 minutes] to complete**. The answers from all people interviewed will be combined and your responses will remain confidential. Participation in the survey is voluntary. You may withdraw from the survey at any time or refuse to answer any questions if you wish **(please refer to information sheet for additional information).**  ***Interviewer note: If you are directed to someone else then please repeat the introduction.*** | Tick boxes against the two options **in black** | Two options **in black** |
|  |  |  |  |  |
|  | 1 | **Program ID** |  | *Automatically generated number* |
|  |  |  |  |  |
| Contact information |  | Before I commence I need to collect your contact information.  ***Interviewer note: you may be able to ask for a business card if face to face or email signature to complete this section or pre-fill*** |  |  |
|  | 2a | What is the name of your organisation? |  | *Text box single line* |
|  | 2b | What is your full name? | First name  Middle name  Surname | *Drop down menu to title*  *Text box single line*  *Text box single line* |
|  | 2c | What is your job or position title? | Free text | *Text box single line* |
|  | 2d | What is your contact phone number? | Phone number  Mobile number ***if appropriate*** | *Text box single line*  *Text box single line* |
|  | 2e | What is your email address? | Email | *Single line textbox field – RULE: must include ‘@’ symbol in response* |
|  | 2f | What is your postal work address? | Postal address fields | *List all fields necessary to collect postal address* |
|  | 2g | What type of organisation to do you work for? | READ OUT:  Names of type of organisations from drop down menu | *Drop down menu using the following categories:*  Local Government (go to Q3)  State Government (go to Q4)  Federal Government (go to Q4)  Not-for-profit (go to Q4)  Community (go to Q4)  Non-government organisation (go to Q4)  Corporate (go to Q4)  Private (go to Q4)  Other (go to Q4)  **Describe other** (Show single line textbox field): (go to Q4) |
|  | 3 | ***Interviewer note: if local government only.***  What is the name of the person in your local government who has the responsibility for public health planning? | First name  Surname  Contact email | *Text box single line*  *Text box single line*  *Text box single line* |
|  |  |  |  |  |
| Programs being conducted | 4 | I am now going to ask you about any programs that you know of in your area that promote **nutrition** or **physical activity** or aim to reduce **overweight or obesity** that are being conducted in your area.  **Please note - We are only interested in programs that are: current (in the last 12 months), conducted in XXXX area, designed to reach a group or population, and is more than a one-off activity.**  ***Interviewer: Aim is to get a list of the types of programs that are being conducted in the area. Following this you can exclude if not appropriate using the inclusion/exclusion criteria listed below (Q9+10)*** |  |  |
| Nutrition | 4a | **Starting with nutrition**….. Do you know of any programs that address nutrition in your area? | Yes **Go to Q4b**  No **Go to Q5a** | *Drop down Yes/no* |
|  | 4b | Can you tell me the names of the nutrition program/s | List name/s of program  **[Interview: after first response probe for other programs]** | *Sub-table* |
|  |  |  |  |  |
| Physical activity | 5a | Do you know of any programs that address physical activity in your area? | Yes **Go to Q5b**  No **Go to Q6a** | *Drop down*  *Yes/no* |
|  | 5b | Can you tell me the name/s of the physical activity programs? | List name/s of program  **[Interview: after first response probe for other programs]** | *Multiple single textbox lines* |
|  |  |  |  |  |
| Overweight or obesity | 6a | **Now to health promotion programs to address *overweight or obesity*.**... Do you know of any programs that address overweight, obesity or healthy-weight in your area? | Yes **Go to Q6b**  No **Go to Q7a** | *Drop down*  *Yes/no* |
|  | 6b | Can you tell me the name/s of the overweight or obesity programs in your area? | List name/s *of programs*  **[Interview: after first response probe for other programs]** | *Sub-table* |
|  |  |  |  |  |
| Responsible organisations | 7a | I’m now going to ask you, of the programs we discussed, which ones are your organisation:   - - **Involved in**     - The organisation is involved in the day to day delivery of the program but no funding or reporting requirements.   - **Responsible for**     - The organisation is responsible for the coordination, funding and/or the administration of the program i.e. responsible for the outcomes and reporting to funders etc. Please re-enter their details in question **7.b** before proceeding to question 8.   - **Neither**     - The organisation is neither responsible for nor involved in the program, yet they know this program exits in their area. Please ensure you complete question **7.b** before proceeding to the end question **19.** | 1) Involved in;  2) Responsible for delivering; or  3) Neither. | - *Tick-box* - *Allow multiple responses for 1 & 2* |
|  | 7b | Are you the person responsible for the delivery of this program? | Yes Go to Q.8  No Go to Q.7c | *Drop down tick box*  *Yes/no* |
|  | 7c | Can you please tell me the name of the person and organisation responsible for delivering this program?  **Interviewer: If unknown, please insert ‘unknown’** | First name  Last name  Organisation | *Single line textbox (First name)*  *Single line textbox (Last name)* |
|  |  |  |  |  |
| Name of program | 8 | I am now going to go through each program you have indicated you are responsible for one by one to see if it meets our audits inclusion/exclusion criteria. |  |  |
| Inclusion criteria | 9a | ***Show physical map of XXXX area.***  Referring to this map, please indicate the suburbs the program is being delivered in: | Include dropdown list of all suburbs located in the:   - [List of suburbs appears here] | *Drop down multiple tick option with list of suburbs below each of the following LGAs:*  *[List of LGAs appears here]* |
|  | 9b | Is it a community or population based program that is not a one-on-one or clinical focussed program? | Yes  No | *Drop down tick box*  *Yes/no* |
|  |  |  |  |  |
|  | 9c | Is this program current? | Yes  No | *Drop down tick box*  *Yes/no* |
|  |  |  |  |  |
|  | 9d | Are two or more program sessions offered? i.e. It is more than a one off session. | Yes  No | *Drop down tick box*  *Yes/no* |
|  |  |  |  |  |
| Exclusion criteria:  Note:  If answer YES to any in Q.10 then exclude) | 10 | Does the program involve a pharmacological intervention that includes the use of drugs or nutrition supplements? | Yes  No | *Drop down tick box*  *Yes/no* |
|  |  | ***Interviewer note:***  ***Now that I have established that the program meets our inclusion/exclusion criteria, I would like to ask some specific questions about the program*** |  |  |
| Detailed description of programs they are responsible for | * | The next section gathers more specific information regarding the programs you offer or are responsible for. |  |  |
|  |  |  |  |  |
|  | 11a | Would you describe the program as an ‘overarching program’ or a ‘sub-program’ or a ‘standalone’ program? | Overarching program  Sub-program program  Standalone program | *Drop down menu with three options and tick box* |
|  | 11b | What is the name of this overarching program? | Name of overarching program | *Free text to list name of program* |
|  |  |  |  |  |
|  | 12a | Is there a website address or Facebook page for this program? | Yes **Go to Q12b**  No **Go to Q13a**  Don’t know **Go to Q13a** | *Drop down tick box with yes/no/don’t know* |
|  |  |  |  |  |
|  | 12b | What is the website address or Facebook page link for the program? | List web address | *Single line text box if known* |
|  |  |  |  |  |
| Aim | 13a | **I am now going to ask about the aim, objectives and strategies for this program.**  What is the main aim or goal of the program? | State main goal/aim | *Multiple line text box* |
| Objectives | 13b | I am going to READ OUT a number of **objectives** of health promotion programs.  Can you please respond yes or no as to whether this program aims to …? ***(Interviewer note: read out)***   1. Raise awareness of the issue 2. Influence attitudes regarding the issue 3. Increase knowledge of the issue 4. Build skills to influence the issue 5. Change behaviour 6. Encourage enrolment in services 7. Advocate for government changes 8. Develop or enforce regulation or legislation 9. Influence changes to the built environment 10. Partnership development 11. Any other aim not captured above | Yes or no  Yes or no  Yes or no  Yes or no  Yes or no  Yes or no  Yes or no  Yes or no  Yes or no  Yes or no  Text box | *Drop down tick box with yes/no/don’t know*  *Plus multi line text box for ‘other’* |
| Strategies | 13c | I am going to READ OUT list of **strategies** that health promotion programs use to achieve their objectives. Which of the following, if any, does your program use….? ***(Interviewer note: read out)***   1. Paid media (either TV, radio, or newspaper) 2. Social media (such as Facebook, twitter) 3. Unpaid media or public relations events (such as local newspaper or events) 4. Online communication (such as websites, emails) 5. Online resources 6. Printed resources 7. Telephone support 8. School curriculum 9. Policy or guidelines 10. Incentives (such as gifts or grants) 11. Group education sessions 12. Referral service 13. Signage 14. Establish partnerships 15. Other, not listed above | Yes or no  Yes or no  Yes or no  Yes or no  Yes or no  Yes or no  Yes or no  Yes or no  Yes or no  Yes or no  Yes or no  Yes or no  Yes or no  Yes or no  Text box | *Drop down menu yes /no for each question*  *Plus multiline free textbox for “Other”* |
| Target audience | 13d | How would you describe the target audience for the program, or in other words, who is the program designed to reach? | Primary target group  Secondary target group | *Two single line textboxes for each ‘Primary’ and ‘Secondary’ fields* |
| Program reach | 13e | How many people would you say this program reaches each year? | Primary target group  Secondary target group | *Single line textbox - numbers*  *Single line textbox - numbers* |
