# Supplementary material for: Initiatives and partnerships in an Australian metropolitan obesity prevention system: a social network analysis
Source: BMC Public Health. 2021 Aug 12;21:1542. doi: 10.1186/s12889-021-11599-7 (PMC8359547; doi:10.1186/s12889-021-11599-7)
Supplement: Supplementary file 2 — Additional File 2: Organisational Network Survey. Organisational network data were collected via an online survey using Qualtrics. Participants were asked to answer a series of network questions for each of the identified organisations (n = 30), which asked about the: relationship with each organisation across several domains (share information or knowledge; share resources; engage in joint planning or run joint programs; receive funding; provide funding; share funding or apply for joint funding) using a scale (high, medium, low, or none). [file 12889_2021_11599_MOESM3_ESM.docx]

**END OF SURVEY**

**Additional File 3**

**Title of data: Prevention system inventory (n=189)**

**Description of data:** This table presents a summary of the objectives, strategies, target groups, setting and funding sources of the initiatives collected during the Systems Inventory.

|  | **Nutrition**  **n=66** | | **Physical activity**  **n=95** | | **Obesity**  **n=28** | | **Total**  **n=189** | |
| --- | --- | --- | --- | --- | --- | --- | --- | --- |
|  | **n** | **%** | **n** | **%** | **n** | **%** | **n** | **%** |
| **Objective** |  |  |  |  |  |  |  |  |
| Influence attitudes | 59 | 89.4 | 74 | 77.9 | 27 | 96.4 | 164 | 86.8 |
| Change behaviour | 58 | 87.9 | 77 | 81.1 | 27 | 96.4 | 162 | 85.7 |
| Increase knowledge | 59 | 89.4 | 74 | 77.9 | 27 | 96.4 | 160 | 84.7 |
| Build skills | 54 | 81.8 | 75 | 78.9 | 27 | 96.4 | 156 | 82.5 |
| Raise awareness | 55 | 83.3 | 70 | 73.7 | 27 | 96.4 | 152 | 80.4 |
| Partnership development | 47 | 71.2 | 63 | 66.3 | 18 | 64.3 | 128 | 67.7 |
| Encourage enrolment | 28 | 42.4 | 58 | 61.1 | 24 | 85.7 | 110 | 58.2 |
| Influence changes to built environment | 21 | 31.8 | 31 | 32.6 | 8 | 28.6 | 60 | 31.7 |
| Advocate change | 12 | 18.2 | 18 | 18.9 | 2 | 7.1 | 32 | 16.9 |
| Develop regulation | 7 | 10.6 | 7 | 7.4 | 4 | 14.3 | 18 | 9.5 |
| **Strategies** |  |  |  |  |  |  |  |  |
| Online communication | 49 | 74.2 | 77 | 81.1 | 24 | 85.7 | 150 | 79.4 |
| Printed resources | 52 | 78.8 | 67 | 70.5 | 25 | 89.3 | 144 | 76.2 |
| Social media | 45 | 68.2 | 67 | 70.5 | 20 | 71.4 | 132 | 69.8 |
| Establish partnerships | 51 | 77.3 | 59 | 62.1 | 19 | 67.9 | 129 | 68.3 |
| Group education sessions | 49 | 74.2 | 53 | 55.8 | 25 | 89.3 | 127 | 67.2 |
| Unpaid media | 35 | 53 | 61 | 64.2 | 20 | 71.4 | 116 | 61.4 |
| Online resources | 44 | 66.7 | 41 | 43.2 | 16 | 57.1 | 101 | 53.4 |
| Telephone support | 30 | 45.5 | 50 | 52.6 | 16 | 57.1 | 96 | 50.8 |
| Referral services | 28 | 42.4 | 42 | 44.2 | 15 | 53.6 | 85 | 45 |
| Signage | 27 | 40.9 | 40 | 42.1 | 10 | 35.7 | 77 | 40.7 |
| Incentives | 29 | 43.9 | 34 | 35.8 | 12 | 42.9 | 75 | 39.7 |
| Policy or guidelines | 15 | 22.7 | 23 | 24.2 | 7 | 25 | 45 | 23.8 |
| Paid media | 8 | 12.1 | 22 | 23.2 | 8 | 28.6 | 38 | 20.1 |
| School curriculum | 7 | 10.6 | 7 | 7.4 | 4 | 14.3 | 18 | 9.5 |
| **Primary target group** |  |  |  |  |  |  |  |  |
| Everyone | 12 | 18.2 | 20 | 21.1 | 2 | 7.1 | 34 | 18 |
| Adults 50 years and over | 5 | 7.6 | 12 | 12.6 | 2 | 7.1 | 19 | 10.1 |
| Workplaces and staff | 5 | 7.6 | 6 | 6.3 | 6 | 21.4 | 17 | 9.0 |
| Low SES and disadvantaged | 10 | 15.2 | 4 | 4.2 | 1 | 3.6 | 15 | 7.9 |
| Youth 12 to 17 years | 3 | 4.5 | 9 | 9.5 | 1 | 3.6 | 13 | 6.9 |
| Families | 4 | 6.1 | 5 | 5.3 | 1 | 3.6 | 10 | 5.3 |
| High risk of chronic disease | 2 | 3.2 | 3 | 3.2 | 5 | 17.9 | 10 | 5.3 |
| Aboriginal and Torres Strait Islander | 4 | 6.1 | 3 | 3.2 | 3 | 10.7 | 10 | 5.3 |
| Adults 18 years and over | 3 | 4.5 | 4 | 4.2 | 0 | 0 | 7 | 3.7 |
| Culturally and linguistically diverse | 0 | 0 | 5 | 5.3 | 1 | 3.6 | 6 | 3.2 |
| People with mental illness | 3 | 4.5 | 1 | 1.1 | 1 | 3.6 | 5 | 2.6 |
| School staff and community | 1 | 1.5 | 1 | 1.1 | 2 | 7.1 | 4 | 2.1 |
| Primary school children 5-12 years | 2 | 3.0 | 2 | 2.1 | 0 | 0 | 4 | 2.1 |
| School aged children 5-17 years | 1 | 1.5 | 2 | 2.1 | 0 | 0 | 3 | 1.6 |
| People living with disability | 0 | 0 | 3 | 3.2 | 0 | 0 | 3 | 1.6 |
| Parents of children below 18 years | 3 | 4.5 | 0 | 0 | 0 | 0 | 3 | 1.6 |
| All children below 18 years | 1 | 1.5 | 2 | 2.1 | 0 | 0 | 3 | 1.6 |
| Community groups | 0 | 0 | 0 | 0 | 2 | 7.1 | 2 | 1.1 |
| Children below 5 years | 0 | 0 | 1 | 1.1 | 0 | 0 | 1 | 0.5 |
| Health professionals | 1 | 1.5 | 0 | 0 | 0 | 0 | 1 | 0.5 |
| **Setting** |  |  |  |  |  |  |  |  |
| Community centre | 31 | 47 | 33 | 34.7 | 9 | 32.1 | 73 | 38.6 |
| Leisure centre | 18 | 27.3 | 30 | 31.6 | 9 | 32.1 | 57 | 30.2 |
| Community garden | 14 | 21.2 | 32 | 33.7 | 7 | 25 | 53 | 28 |
| State-wide | 19 | 28.8 | 7 | 7.4 | 4 | 14.3 | 30 | 15.9 |
| Workplace | 9 | 13.6 | 10 | 10.5 | 9 | 32.1 | 28 | 14.8 |
| Primary school | 14 | 21.1 | 7 | 7.4 | 5 | 17.9 | 26 | 13.8 |
| Secondary school | 11 | 16.7 | 6 | 6.3 | 3 | 10.7 | 20 | 10.6 |
| Government institution | 9 | 13.6 | 2 | 2.1 | 4 | 14.3 | 15 | 7.9 |
| Pre-school | 7 | 10.6 | 2 | 2.1 | 3 | 10.7 | 12 | 6.3 |
| Educational institution | 3 | 4.5 | 2 | 2.1 | 6 | 21.4 | 11 | 5.8 |
| Play group | 4 | 6.1 | 1 | 1.1 | 2 | 7.1 | 7 | 3.7 |
| Day-care centre | 2 | 3.0 | 1 | 1.1 | 2 | 7.1 | 5 | 2.6 |
| **Funding source** |  |  |  |  |  |  |  |  |
| State government | 11 | 16.7 | 9 | 9.5 | 6 | 21.4 | 26 | 13.8 |
| Local government | 8 | 12.1 | 14 | 14.7 | 1 | 3.6 | 23 | 12.2 |
| Charities | 3 | 4.5 | 2 | 2.1 | 0 | 0 | 5 | 2.6 |
| Philanthropy | 3 | 4.5 | 1 | 1.1 | 1 | 3.6 | 5 | 2.6 |
| Federal government | 1 | 1.5 | 1 | 1.1 | 2 | 7.1 | 4 | 2.1 |
