# Supplementary material for: Initiatives and partnerships in an Australian metropolitan obesity prevention system: a social network analysis
Source: BMC Public Health. 2021 Aug 12;21:1542. doi: 10.1186/s12889-021-11599-7 (PMC8359547; doi:10.1186/s12889-021-11599-7)
Supplement: Supplementary file 3 — Additional File 3: Prevention system inventory (n = 189): This table presents a summary of the objectives, strategies, target groups, setting and funding sources of the initiatives collected during the Systems Inventory. [file 12889_2021_11599_MOESM2_ESM.docx]

**Additional File 2**

**Title of instrument: Organisational Network Survey**

**Description of instrument:** Organisational network data were collected via an online survey using Qualtrics. Participants were asked to answer a series of network questions for each of the identified organisations (n=30), which asked about the: relationship with each organisation across several domains (share information or knowledge; share resources; engage in joint planning or run joint programs; receive funding; provide funding; share funding or apply for joint funding) using a scale (high, medium, low, or none).

**Organisational Network Survey**

This survey will ask about how your organisation, as a whole, works with other organisations in undertaking nutrition, physical activity and overweight and obesity prevention in the geographical area of XXXX (map shown below). If you are the suitable person to answer this survey on behalf of your organisation, and the nutrition, physical activity and overweight and obesity activities you conduct, please click below that you agree. If you are unable to complete this survey please select no, and the research team will contact you to identify the person most suitable.

- Yes, I agree.
- No.

Please select the organisation you work for from the drop-down list below.

[List of 30 organisation names]

Does your organisation fall within a larger organisational body? For example, is your organisation the state branch of a national health agency?

- Yes
- No

Yes. If yes, what is the name of the larger organisation your organisation is a part of? ________________________________________________

What is your job title?

________________________________________________________________

How long have you been working for this organisation? (Approximate number of years and/or months)

_______________________________________________________________

|  |
| --- |

How many people in your organisation work in nutrition, physical activity and overweight and obesity prevention?  (Approximate number of full time equivalent staff)

________________________________________________________________

|  |
| --- |

How long has your organisation been in operation? (Approximate number of years)

_______________________________________________________________

|  |  |
| --- | --- |

Please select all of the organisations located in or delivering services within the geographical area of XXXX, that you are aware of as a result of your work in nutrition, physical activity and overweight and obesity prevention, from the list below. Please do not select the organisation you currently work for.

[List of 30 organisations]

The following questions explore the relationships between some organisations located in, or delivering services in the geographical area of XXXX (map shown below), and the different ways they might work with each other. For each question please consider how as an organisation you work with each of the other organisations on the list.

PLEASE ANSWER ON BEHALF OF YOUR ORGANISATION
 
[Map appears here]

1. For each of the organisations listed below, who are located in or delivering services within the geographical area of the XXXX, please indicate the strength of the current relationship (high, medium, low, none), with regards to sharing information or knowledge. This might include talking about opportunities or issues in the local area, or passing on information about events or clients.

- High - A strong organisational relationship, where you regularly share information or knowledge with one another.
- Medium - A common or moderate organisational relationship, where you occasionally share information or knowledge with one another.
- Low - A weak organisational relationship, where you rarely share information or knowledge with one another.

|  | High | Medium | Low | None |
| --- | --- | --- | --- | --- |
| List of 30 organisations (one per row) |  |  |  |  |

2. For each of the organisations listed below, who are located in, or delivering services within the geographical area of the XXXX, please indicate the current strength of the relationship (high, medium, low, none), with regards to how you share resources. This might include in-kind support, sharing staffing, sharing venues, providing training.

- High - A strong organisational relationship, where you regularly share resources with one another.
- Medium - A common or moderate organisational relationship, where you occasionally share resources with one another.
- Low - A weak organisational relationship, where you rarely share resources with one another.

|  | High | Medium | Low | None |
| --- | --- | --- | --- | --- |
| List of 30 organisations (one per row) |  |  |  |  |

For each of the organisations listed below, who are located in, or delivering services in the geographical area of the XXXX, please indicate the current strength of the relationship (high, medium, low, none), with regards to how your organisation engages in joint planning, or in running joint programs. This could include being a part of a committee involved in planning or programming, or in co-ordinating efforts with other organisations.

- High - A strong organisational relationship, where you regularly engage in joint planning/programs with one another.
- Medium - A common or moderate organisational relationship, where you occasionally engage in joint planning/programs with one another.
- Low - A weak organisational relationship, where you rarely engage in joint planning/programs with one another.

|  | High | Medium | Low | None |
| --- | --- | --- | --- | --- |
| List of 30 organisations (one per row) |  |  |  |  |

4. For each of the organisations listed below, who are located in, or delivering services in the geographical area of the XXXX, please indicate the current strength of the relationship (high, medium, low, none), in terms of how your organisation receives funding from each organisation (if any).

- High - A strong organisational relationship, where you receive a significant amount of funding from the organisation.
- Medium - A common or average organisational relationship, where you receive a moderate amount of funding from the organisation.
- Low - A weak organisational relationship, where you receive a minimal amount of funding from the organisation.

|  | High | Medium | Low | None |
| --- | --- | --- | --- | --- |
| List of 30 organisations (one per row) |  |  |  |  |

5. For each of the organisations listed below, who are located in, or delivering services in the geographical area of the XXXX, please indicate the current strength of the relationship (high, medium, low, none), in terms of how your organisation provides funding to each of the organisations (if any).

- High - A strong organisational relationship, where you provide a significant amount of funding to the organisation.
- Medium - A common or average organisational relationship, where you provide a moderate amount of funding to the organisation.
- Low - A weak organisational relationship, where you provide a minimal amount of funding to the organisation.

|  | High | Medium | Low | None |
| --- | --- | --- | --- | --- |
| List of 30 organisations (one per row) |  |  |  |  |

6. For each of the organisations listed below, who are located in, or delivering services in the geographical area of the XXXX, please indicate the current strength of the relationship (high, medium, low, none), with regards to how your organisation shares funding or applies for joint funding with each of the organisations (if any).

- High - A strong organisational relationship, where you share a significant amount of funding between organisations.
- Medium - A common or average organisational relationship, where you share a moderate amount of funding between organisations.
- Low - A weak organisational relationship, where you share a minimal amount of funding between organisations.

|  | High | Medium | Low | None |
| --- | --- | --- | --- | --- |
| List of 30 organisations (one per row) |  |  |  |  |

7. How long has your organisation worked with each of the organisations listed below, who are currently located in, or delivering services in the geographical area of the XXXX, regardless of the capacity of that work?

|  | Short-term  (<6 months) | Medium-term  (6 months to 2 years) | Long-term  (2+ years) | Not at all |
| --- | --- | --- | --- | --- |
| List of 30 organisations (one per row) |  |  |  |  |

8. From your organisational perspective, how frequently does your organisation interact with each of the organisations listed below, who are located in, or delivering services in the geographical area of the XXXX, whether it be face-to-face, online, by mail, email or phone?

|  | Daily | Weekly | Monthly | Quarterly | Biannually | Yearly | Never |
| --- | --- | --- | --- | --- | --- | --- | --- |
| List of 30 organisations (one per row) |  |  |  |  |  |  |  |

9. Please select below, all relevant barriers your organisation experiences implementing nutrition, physical activity and overweight and obesity prevention in the geographical area of the XXXX, if any.

*Please tick all that apply.*

- Limited funding
- Limited 'in kind' resources
- Limited staffing
- Not enough volunteers/volunteer staff
- Limited overweight and obesity prevention expertise
- Limited nutrition expertise
- Limited physical activity expertise
- Insufficient collaborations and partnerships
- No formal health policy
- Political feasibility/amenability to prevention and health promotion
- Food industry interference
- Not enough management support
- Limited expertise of fields other than health
- Insufficient community connections
- Regular staff turnover
- Limited capacity of partner organisations
- Poor facilitation/leadership
- Limited training opportunities
- Not enough evidence/outcomes of successful programs
- Limited advocacy capabilities
- Limited IT/web resources
- Other (please state) ________________________________________________
- Other (please state) ________________________________________________
- Other (please state) ________________________________________________

10. Please rank the following options from highest to lowest, regarding what you believe is the most important current contribution your organisation makes towards improving nutrition, physical activity and overweight and obesity prevention in the geographical area of the XXXX. To answer, please click and drag the responses to order them from highest to lowest contribution, 1 being the highest and 10 being the lowest.

______ Funding

______ In kind resources

______ Paid staff

______ Volunteers/volunteer staff

______ Specific overweight and obesity expertise

______ Specific nutrition expertise

______ Health and prevention data

______ Program development and evaluation

______ Specific physical activity expertise

______ Developing health policy

______ Expertise other than health

______ Program support and coordination

______ Community connections

______ Facilitation/leadership

______ Advocacy

______ IT/web resources

______ Other (please state)

______ Other (please state)

______ Other (please state)

______ Other (please state)

11. Please select below how frequently your organisation uses any of the following resources to disseminate information.

|  | Daily | Weekly | Monthly | Quarterly | Biannually | Yearly | Never |
| --- | --- | --- | --- | --- | --- | --- | --- |
| Media (for example television, radio, print, etc.) |  |  |  |  |  |  |  |
| Social media (for example Facebook, Twitter, LinkedIn, etc.) |  |  |  |  |  |  |  |
| Blogs |  |  |  |  |  |  |  |
| Websites |  |  |  |  |  |  |  |
| Newsletters/e-newsletters |  |  |  |  |  |  |  |
| Subscription lists or listservs |  |  |  |  |  |  |  |
| Reports |  |  |  |  |  |  |  |
| Peer-reviewed publications |  |  |  |  |  |  |  |
| Attending conferences |  |  |  |  |  |  |  |
| Professional development (for example webinars, workshops, information sessions, etc.) |  |  |  |  |  |  |  |
| Meetings outside of your organisation |  |  |  |  |  |  |  |
| Communications with colleagues and experts (for example emails or informal discussion) |  |  |  |  |  |  |  |
| Other (please state) |  |  |  |  |  |  |  |
| Other (please state) |  |  |  |  |  |  |  |
| Other (please state) |  |  |  |  |  |  |  |

12. Please select below how frequently your organisation uses any of the following resources to obtain information.

|  | Daily | Weekly | Monthly | Quarterly | Biannually | Yearly | Never |
| --- | --- | --- | --- | --- | --- | --- | --- |
| Media (for example television, radio, print, etc.) |  |  |  |  |  |  |  |
| Social media (for example Facebook, Twitter, LinkedIn, etc.) |  |  |  |  |  |  |  |
| Blogs |  |  |  |  |  |  |  |
| Websites |  |  |  |  |  |  |  |
| Newsletters/e-newsletters |  |  |  |  |  |  |  |
| Subscription lists or listservs |  |  |  |  |  |  |  |
| Reports |  |  |  |  |  |  |  |
| Peer-reviewed publications |  |  |  |  |  |  |  |
| Attending conferences |  |  |  |  |  |  |  |
| Professional development (for example webinars, workshops, information sessions, etc.) |  |  |  |  |  |  |  |
| Meetings outside of your organisation |  |  |  |  |  |  |  |
| Communications with colleagues and experts (for example emails or informal discussion) |  |  |  |  |  |  |  |
| Other (please state) |  |  |  |  |  |  |  |
| Other (please state) |  |  |  |  |  |  |  |
